# Reduced Expression of TET1, TET2, TET3 and TDG mRNAs Are Associated with Poor Prognosis of Patients with Early Breast Cancer

**DOI:** 10.1371/journal.pone.0133896

**Published:** 2015-07-24

**Authors:** Liu Yang, San-Jian Yu, Qi Hong, Yu Yang, Zhi-Ming Shao

**Affiliations:** 1 Department of Breast Surgery, Fudan University Shanghai Cancer Center; Department of Oncology, Shanghai Medical College, Fudan University, shanghai, China; 2 Institutes of Biomedical Sciences, Fudan University, Shanghai, P.R. China; 3 School of basic medical sciences, Chengdu University of traditional Chinese medicine, Chengdu, P.R. China; The University of Hong Kong, CHINA

## Abstract

**Purpose:**

The purpose of this study was to determine the prognostic role of ten eleven translocation (TET) family proteins and DNA glycosylase (TDG) in patients with early breast cancer (EBC).

**Methods:**

Expression of mRNAs encoding TET1–3 and TDG in 162 breast cancer tissues was quantified using real-time polymerase chain reaction analysis. The general characteristics of patients and clinicopathologic factors were collected. Estimation of patient survival was calculated using the Kaplan–Meier method, and independent prognostic indicators were analyzed using Cox regression analysis.

**Results:**

The level of TET1 mRNA was significantly related to overall survival (OS) (P = 0.022). Multivariate analysis shows that the TNM stage was an independent predictor of disease-free survival (DFS) (HR = 1.761, 95% CI: 1.124–2.761, P = 0.014) and OS (HR = 2.135, 95% CI: 1.070–4.263, P = 0.032). Further, in patients with EBC who were treated with anthracyclines, Kaplan–Meier analysis indicates that the levels of TET3 and TDG mRNAs were related to DFS (P = 0.026 and 0.030, respectively), and multivariate analysis reveals that high levels of TET3 (HR = 1.944, 95% CI: 1.029–3.672, P = 0.040) and TDG (HR = 2.178, 95% CI: 1.140–4.163, P = 0.018) mRNAs were independent indicators of favorable DFS.

**Conclusions:**

Our study indicates that EBC patients with decreased expression of TET1 mRNA had worse OS and that the levels of TET3 and TDG mRNAs were independent prognostic factors for patients who received anthracycline chemotherapy.

## Introduction

Breast cancer is one of the most commonly diagnosed cancers in women, with an estimated 1.2 million new cases worldwide each year, and represents approximately 25% of cancers of women [1, 2]. Patients respond well to treatment, and standard guideline has been promoted in our country in recent years; however, breast cancer remains the second most frequent cause of cancer-related deaths, and 1.2 million people die each year in our country [3, 4]. The development of new techniques to predict the prognosis of breast cancer is crucial for administering more timely and appropriate treatment, and further research is required to identify novel molecular markers of prognosis.

The influence of epigenetics has contributed to the understanding of the complexities of gene regulation, cell differentiation, aging, and disease [5–8], and aberrant epigenetic profiles are associated with the pathogenesis of cancer [9, 10]. For example, methylation of promoter regions leads to epigenetic gene silencing, particularly methylation of cytosine residues in CpG islands [11], which is a short stretch of DNA with higher frequency of the CG sequence than other regions, locating around the promoters of housekeeping genes or other genes frequently expressed in a cell. And further, dysregulated DNA methylation of tumor suppressor genes occurs in different types of cancer [12]. Marc Milstein[13] reported that RIN1 gene was silenced in breast tumor cell lines compared to cultured human mammary epithelial cells and DNA methylation within the RIN1 promoter contributed to silence of the gene.

Conversely, DNA demethylation occurs in different biological contexts, and this alteration can occur passively or actively [14]. Passive DNA demethylation refers to the loss of 5-methylcytosine (5mC) residues by gradual dilution in a replication-dependent manner. The active process involves TET family proteins and TDG. Oxidation of 5mC to 5-hydroxymethylcytosine (5hmC) by TET proteins is the key process of active DNA demethylation. Further oxidation of 5hmC by TET generates 5-formylcytosine (5fC) and 5-carboxylcytosine (5caC), which can be actively removed from the genome by TDG [15, 16].

The TET family of DNA dioxygenases TET1, TET2, and TET3 require α-ketoglutarate and Fe^2+^ for activity [14]. Loss-of-function mutations or decreased expression of TETs and TDG inhibits the DNA demethylation pathway, which prevents the removal of 5mC from genomic DNA. And aberrant methylation of tumor suppressor genes may lead to tumorigenesis. TET2 mutations, such as gene deletion, occur in chronic myelomonocytic leukemia, acute myeloid leukemia, and myelodysplastic syndromes as well as in solid tumors, including clear-cell renal cell carcinoma, prostate cancer, and breast cancer [17–21]. Moreover, mutations of the three TET genes were detected in colorectal cancer [22], and there is a close correlation between TET expression and robust tumor growth and metastasis [23–25]. Yang et al. [26] reported that the levels of 5hmC are dramatically reduced in human breast cancer and that the expression of the three TET genes was significantly reduced in breast cancer, particularly that of TET1. They further found that 5hmC levels are broadly decreased in breast cancer tissues and tightly linked with tumorigenesis. Therefore, detection of 5hmC may serve as a valuable biomarker for the diagnosis of breast cancer. Further more, Hsu [25] demonstrated the inhibition of the invasiveness of breast cancer cells by TET1 in vivo and that down-regulation of TET1 expression in patients with breast cancer correlates with poor survival. The proof whether other proteins as well as TDG may serve as prognostic markers of breast cancer is lacking. In this present study, we further analyzed the association between the expression of mRNAs encoding TET1–3 and TDG with the prognosis of breast cancer patients.

## Materials and Methods

### Patients and samples

The present study included 162 patients who were diagnosed with early breast cancer (EBC) according to histopathological analyses conducted at Shanghai Cancer Center, Fudan University from April 2002 to November 2005. The early breast cancer patients in this cohort indicated those patients who can be treated with operation, namely early operable breast cancer. Breast cancer tissues were received immediately after surgery and stored at −80°C. The expression levels of estrogen receptor (ER), progesterone receptor (PR), and human epidermal growth factor-2 (HER-2) status were determined by the Department of Pathology of Shanghai Cancer Center, Fudan University according to the guidelines of the American Society of Clinical Oncology/College of American Pathologists (ASCO/CAP) [27, 28]. Anthracycline is one of the chemotherapeutics and are commonly combined with platinum drugs in adjuvant therapy of breast cancer. We selected patients treated with commonly used anthracyclines to determine their prognoses as a function of expression levels of TET and TDG mRNAs.

### RNA isolation and RT-PCR

Total RNA was extracted from breast cancer tissues using TRIZOL reagent (Invitrogen, Thermo Fisher Scientific Inc. Waltham, MA USA) and complementary DNA was synthesized using RT PCR kit (TOYOBO CO., LTD. Osaka, Japan) according to the manufacturer’s instructions.

### Quantitative real-time PCR

PCR amplification was performed at 95°C for 60 s followed by 40 cycles at 95°C for 15 s, 60°C for 15 s, and 72°C for 45 s using an Applied Biosystems 7900HT Fast Real-Time PCR System (Applied Biosystems, Foster City, CA) with 2.0 μl of cDNA and SYBR Green Real-time PCR Master Mix (Toyobo). Data were collected and analyzed using SDS2.4 Software (Applied Biosystems). Primer sequences are shown in Table 1.

**Table 1:** Primers used for real-time PCR analysis.

Gene	Forward primer (5′–3′)	Reverse primer (5′–3′)
TET1	CAGAACCTAAACCACCCGTG	TGCTTCGTAGCGCCATTGTAA
TET2	GATAGAACCAACCATGTTGAGGG	TGGAGCTTTGTAGCCAGAGGT
TET3	TCCAGCAACTCCTAGAACTGAG	AGGCCGCTTGAATACTGACTG
TDG	TGAAGCTCCTAATATGGCAGTTG	TTCCACTGGTTGTTTTGGTTCT
GAPDH	GCCTCAAGATCATCAGCAATGCCT	TGTGGTCATGAGTCCTTCCACGAT

### Statistical analysis

Statistical analysis was performed using SPSS16.0 software. The levels of mRNAs encoding TETs and TDG were classified as high or low using a cutoff value equal to the median value of all patients’ samples. DFS and OS were calculated using the Kaplan–Meier method, and the log-rank test was used to determine the significance of differences between OS or DFS rates and the expression levels of mRNAs encoding TETs and TDG. P < 0.05 was considered statistically significant. Multivariate analysis was performed using the Cox regression model.

### Ethical statement

The protocol for the use of human tissues was reviewed and approved by the Medical Ethics

Committee of Cancer Hospital, Fudan University (Shanghai, China). Prior to the study, all patients gave their written informed consent to allow us to use leftover tissue samples for scientific research.

## Results

### Clinicopathological characteristics of patients

The median age of the 162 patients was 52 years (range, 33–84 years), and ages were distributed as follows: n = 130 (80.2%) >45 years; n = 32 (19.8%) <45 years. Ninety-three (57.4%) patients were postmenopausal and 69 (42.6%) were premenopausal. The sizes of tumors were as follows: 114 (70.4%) >2 cm and 48 (29.6%) <2 cm. Lymph node metastasis was detected in 71 (43.8%) patients. Disease stages (TNM) were as follows: n = 29 (17.9%), stage I; n = 102 (63.0%), stage II; and n = 31 (19.1%), stage III. Detection of ER, PR, and HER-2 expression was as follows: n = 69 (42.6%), n = 66 (40.7%), and n = 36 (22.2%) (Table 2).

**Table 2:** Clinicopathological characteristics of patients.

Number of patients	162	
Median age at diagnosis ± SD	52 ± 10.4	
Age distribution (years)		
<45	32	19.8%
≥45	130	80.2%
Menopausal status		
Premenopausal	69	42.6%
Postmenopausal	93	57.4%
Tumor size (cm)		
≤2	48	29.6%
>2	114	70.4%
Lymph node status		
Negative	91	56.2%
Positive	71	43.8%
TNM stage		
I	29	17.9%
II	102	63.0%
III	31	19.1%
ER status		
Negative	93	57.4%
Positive	69	42.6%
PR status		
Negative	96	59.3%
Positive	66	40.7%
HER-2 status		
Negative	108	66.7%
Positive	36	22.2%
NA	18	11.1%

### Analysis of expression levels of TET1 mRNA and OS

**Fig 1:**
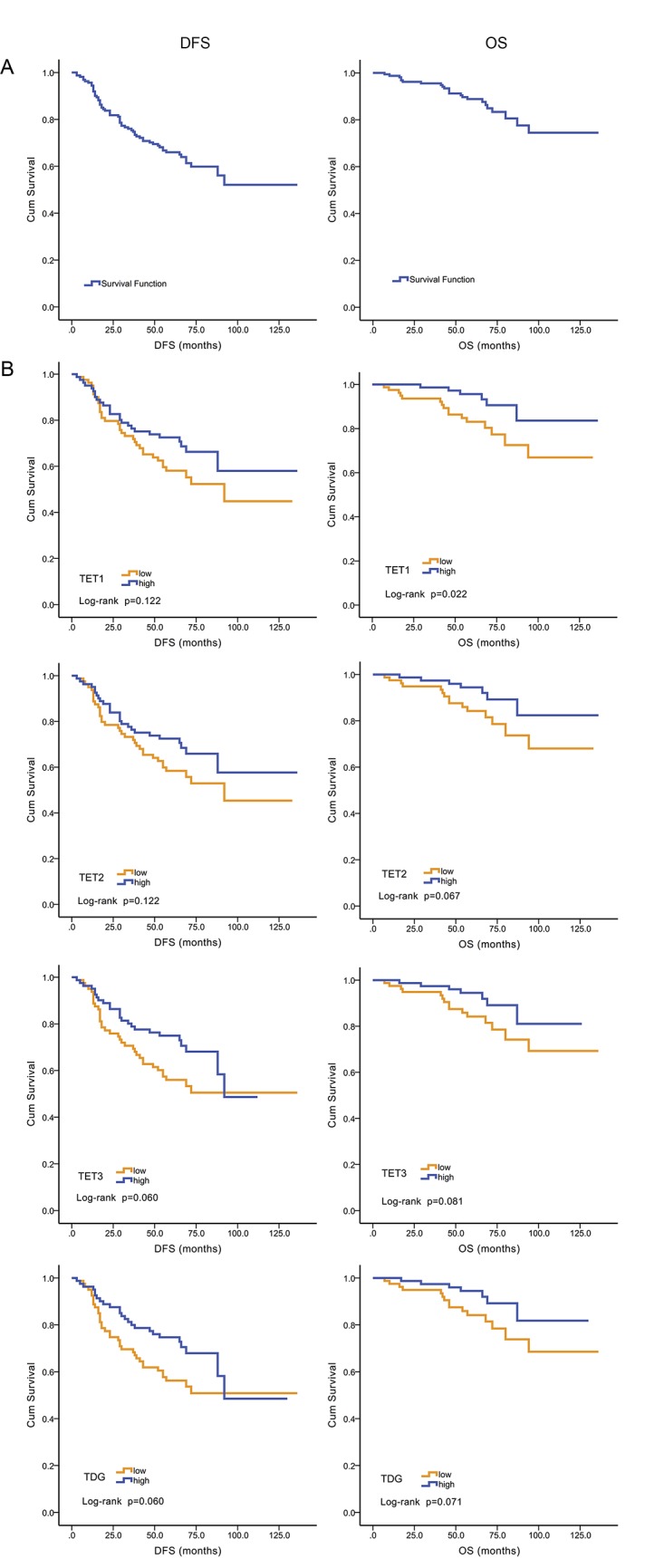
Kaplan–Meier analysis of DFS and OS as a function of TET1 mRNA levels. A. The DFS and OS of all patients were 0.521 and 0.745, respectively. B. Correlation between TET1-3 and TDG mRNAs with DFS and OS in 162 EBC patients. Higher level of TET1 mRNA was related to better OS (P = 0.022) (log-rank test).

The DFS and OS of all patients were 0.521 and 0.745 (Fig 1A), respectively. When patients were stratified according to high or low levels of TET1 expression, DFS was 0.580 or 0.448, respectively (P = 0.122), and OS was 0.836 or 0.669, respectively (P = 0.022) (Fig 1B). Prognosis was better for patients with high levels of TET2, TET3, and TDG mRNAs, although the differences were not statistically significant (P = 0.060–0.122). Further, there was no significant correlation between the expression of any of the mRNAs with clinicopathological parameters such as TNM stage, ER, PR, and HER-2 status.

### Evaluation of TNM stage as an independent prognostic factor for all patients

Multivariate analysis of DFS and OS indicated that TNM stage correlated with patients’ DFS (HR = 2.135, 95% CI: 1.070–4.263, P = 0.032) and OS (HR = 1.761, 95% CI: 1.124–2.761, P = 0.014) (Table 3), indicating that TNM stage was an independent factor for patients’ prognoses.

**Table 3:** Multivariate analysis of DFS and OS in patients with EBC.

	DFS		OS	
	HR (95% CI)	P value	HR (95% CI)	P value
Age (years)	0.973 (0.504–1.878)	0.935	1.712 (0.487–6.020)	0.402
TNM	1.761 (1.124–2.761)	0.014	2.135 (1.070–4.263)	0.032
ER	0.730 (0.414–1.285)	0.275	0.507 (0.198–1.302)	0.158
PR	0.969 (0.550–1.707)	0.912	1.277 (0.507–3.214)	0.604
HER-2	1.335 (0.924–1.929)	0.124	0.642 (0.279–1.481)	0.299
TET1	0.757 (0.235–2.443)	0.641	0.195 (0.031–1.246)	0.084
TET2	1.095 (0.325–3.690)	0.884	2.597 (0.352–19.172)	0.349
TET3	0.856 (0.361–2.027)	0.724	0.960 (0.219–4.216)	0.957
TDG	0.786 (0.415–1.489)	0.461	0.651 (0.223–1.90)	0.432

### Comparison of TET3 and TDG mRNA levels with OS of patients treated with anthracyclines

**Fig 2:**
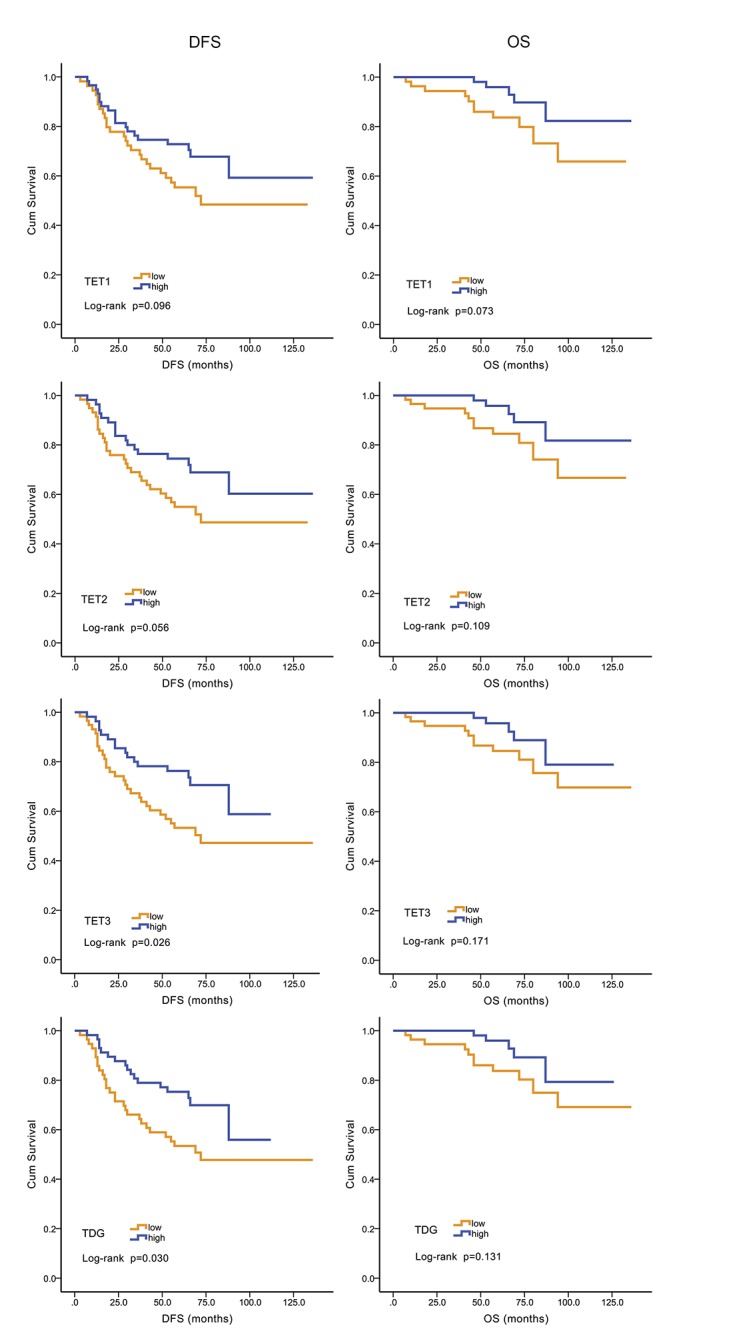
Kaplan–Meier analysis of DFS and OS as a function of TET3 and TDG expression. The levels of TET3 and TDG mRNAs correlated inversely with DFS in patients treated with anthracyclines. Higher levels of TET3 and TDG mRNAs correlated with better DFS (P = 0.026 and 0.030, respectively).

After surgery, 114 (70%) patients received a chemotherapy regimen that included anthracyclines. Kaplan–Meier analysis indicated that higher levels of TET3 (P = 0.026) and TDG (P = 0.030) mRNAs associated with better DFS, but the association between TET3 (P = 0.171) and TDG (P = 0.131) mRNA levels and OS was not statistically significant. DFS and OS did not correlate significantly with TET1 or TET2 mRNA levels (P = 0.056–0.109) (Fig 2).

### Evaluation of TET3 and TDG mRNA levels as independent prognostic factors for patients treated with anthracyclines

Multivariate analysis revealed that high levels of TET3 (HR = 1.944, 95% CI: 1.029–3.672, = 0.040) and TDG (HR = 2.178, 95% CI: 1.140–4.163, P = 0.018) mRNAs were independent indicators of favorable DFS (Table 4).

**Table 4:** Multivariate analysis of DFS and OS in patients treated with anthracyclines.

	DFS		OS	
	HR (95% CI)	P value	HR (95% CI)	P value
TET1	1.653 (0.880–3.108)	0.118	2.682 (0.850–8.460)	0.092
TET2	1.817 (0.964–3.422)	0.065	2.249 (0.717–7.058)	0.165
TET3	1.944 (1.029–3.672)	0.040	1.902 (0.617–5.867)	0.263
TDG	2.178 (1.140–4.163)	0.018	1.902 (0.639–5.665)	0.248

## Discussion

Aberrant methylation of tumor suppressor genes is a hallmark of cancer pathogenesis and is caused by the dysregulation of DNA methylation and demethylation. TET family and TDG proteins represent key factors in the active DNA demethylation pathway. Moreover, a loss-of-function mutation in the TET2 gene is associated with hematological malignancies [18], and mutations in the three TET genes are related to solid tumors [22]. Hsu [25] reported an association between decreased 5hmC levels and TET expression in cancers. However, further investigations of the value of TET and TDG expression levels require further investigation.

In the present study, we analyzed patients’ clinicopathological characteristics and the levels of mRNAs encoding TET1–3 and TDG proteins that were present in tumor tissue. We found that TET1 expression closely correlated with OS. Hsu [25] reported that TET1 mRNA expression correlates inversely with the survival of patients with breast cancer patient and that down-regulation of TET expression correlates positively correlated with larger tumor size and advanced stage. We show here that OS was longer for patients with EBC with high levels of TET1 mRNA, which is consistent with Hsu’s data. There was a similar trend for TET2, 3 and TDG expression, although the differences were not statistically significant. This may be explained by insufficient number of samples. Therefore, we hypothesized that expression level of TET2, 3 and TDG mRNAs may be associated with patients’ prognosis. However, Cox regression analysis did not indicate TET1, 2, 3 and TDG mRNAs were independent predictors of breast cancer. Consistent with the results of other studies [29], we show here that TNM stage predicts prognosis of patients with EBC.

Anthracyclines are one of the most important and commonly used drugs for treating patients with breast cancer. We choose different chemotherapy regimens according to pathological types of breast tumors. Moreover, sensitivity to chemotherapy differs according to the different subtypes. Therefore, we asked whether the expression levels of the four mRNAs studied here were associated with patients’ responses to chemotherapy and found that higher levels of TET3 and TDG mRNAs were associated with improved survival of patients treated with anthracyclines after surgery and served as independent prognostic factors. These findings indicate that patients who express high levels of TET3 and TDG mRNAs may benefit from further chemotherapy and that treatment with regimens including anthracyclines might be a good choice. However, the reliability and reproducibility of our findings require further study.

The results of our study are slightly limited by the proportion of patients in the cohort. Proportion of ER- or PR- patients were higher than that in general breast cancer patients. This might because these patients were diagnosed and treated in a certain time interval in Fudan University Shanghai Cancer Center, during which proportion of ER-/PR- patients were relatively high among all patients.

In summary, our study demonstrates that patients with breast cancer with high levels of TET1 mRNA had better OS than those with low expression of TET1 and that TNM stage was a prognostic factor. Further, the levels of TET3 and TDG mRNAs may serve to predict patients’ responses to anthracyclines and that the DFS of patients with high levels of TET3 and TDG mRNAs may be improved by treatment with anthracyclines.
